# Supplementary material for: Factors contributing to low tuberculosis diagnosis among children aged 0–14 years in Gem Sub County in Siaya County, Kenya
Source: PLoS One. 2026 Mar 6;21(3):e0335326. doi: 10.1371/journal.pone.0335326 (PMC12965594; doi:10.1371/journal.pone.0335326)
Supplement: Attachment — Submitted filename: PONE-D-25-53931_reviewer.docx [file pone.0335326.s001.docx]

PLOS ONE

Factors Contributing to Low Tuberculosis Diagnosis Among Children Aged 0-14 Years in Gem Sub County in Siaya County, Kenya.

--Manuscript Draft--

| Manuscript Number: | PONE-D-25-53931 |
| --- | --- |
| Article Type: | Research Article |
| Full Title: | Factors Contributing to Low Tuberculosis Diagnosis Among Children Aged 0-14 Years in Gem Sub County in Siaya County, Kenya. |
| Short Title: | Low Tuberculosis Diagnosis Among Children Aged 0-14 Years in Gem Sub County in Siaya County, Kenya. |
| Corresponding Author: | Lilian Atieno Okumu, MPH  Jaramogi Oginga Odinga University of Science and Technology Bondo, KENYA |
| Keywords: | Tuberculosis, diagnosis, Paediatrics, Siaya, Kenya |
| Abstract: | Tuberculosis (TB) is a top global health challenge, with 1.2 million children aged 0–14 years becoming ill with TB every year. Yet, a significant proportion remain undiagnosed or missed due to diagnostic barriers. This cross-sectional mixed methods study investigated factors contributing to low tuberculosis diagnosis among children in Gem Sub County, Siaya County Kenya, a high burden region. Data was collected from 71 healthcare workers (HCWs) and 16 respondents across six wards using semistructured questionnaires, key informant interviews, and TB register abstraction. Qualitative data underwent deductive thematic analysis while quantitative data was analyzed using descriptive statistics and logistic regression in SPSS version 27. About a third (31.3%) of TB cases in children required three or more facility visits before diagnosis, with some needing up to 12 visits. Costly and inaccessible chest X-rays and GeneXpert underutilization due to difficulties in sputum and alternative sample (gastric lavage, nasopharyngeal aspirates) collection were key diagnostic constraints.  Delayed care-seeking due to stigma, misconceptions (56.3% linked childhood TB to HIV) and low TB symptom awareness (37.5% of children presented with ≤2 symptoms) were patient related factors associated with low TB diagnosis. About 56.3% of caregivers sought routine TB screening, but 62.5% sought care only after persistent symptoms. Clinical officers were more confident in sample collection (OR=34, 95% CI 3.81–303.21, p=0.002) and GeneXpert interpretation (OR=15, 95% CI 3.20–70.39, p<0.001) than nurses. Trained HCWs were 7.16 times more likely to interpret X-rays accurately (95% CI 2.16–23.67, p<0.001). Enhanced HCW training, improved diagnostic access, targeted community education on stigma and misconceptions are critical for early TB detection in children. |
| Order of Authors: | Lilian Atieno Okumu, MPH |
|  | Daniel Ogungu Onguru, PhD |
|  | David Otieno Odongo, PhD |
|  | James Onyuro Oketch, MSc. |
| Additional Information: |  |
| Question | Response |
| Financial Disclosure  Enter a financial disclosure statement that describes the sources of funding for the work included in this submission. Review the [submission guidelines](http://journals.plos.org/plosone/s/submission-guidelines#loc-financial-disclosure-statement) for detailed requirements. View published research articles from [PLOS ONE](https://journals.plos.org/plosone/) for specific | The author(s) received no specific funding for this work. |

| examples.  This statement is required for submission and will appear in the published article if the submission is accepted. Please make sure it is accurate. |  |
| --- | --- |
| Funded studies Enter a statement with the following details: - Initials of the authors who received each award - Grant numbers awarded to each author - The full name of each funder - URL of each funder website - Did the sponsors or funders play any role in the study design, data collection and analysis, decision to publish, or preparation of the manuscript? |  |
| Did you receive funding for this work? |  |
| Competing Interests  Use the instructions below to enter a competing interest statement for this submission. On behalf of all authors, disclose any [competing interests](http://journals.plos.org/plosone/s/competing-interests) that could be perceived to bias this work—acknowledging all financial support and any other relevant financial or nonfinancial competing interests.  This statement is required for submission and will appear in the published article if the submission is accepted. Please make sure it is accurate and that any funding sources listed in your Funding Information later in the submission form are also declared in your Financial Disclosure statement.  View published research articles from [PLOS ONE](http://journals.plos.org/plosone/) for specific examples. | The authors have declared that no competing interests exist |

| NO authors have competing interests Enter: The authors have declared that no competing interests exist. Authors with competing interests Enter competing interest details beginning with this statement: I have read the journal's policy and the authors of this manuscript have the following competing interests: [insert competing interests here] |  |
| --- | --- |
| * typeset |  |
| Ethics Statement  Enter an ethics statement for this submission. This statement is required if the study involved:   - Human participants - Human specimens or tissue - Vertebrate animals or cephalopods - Vertebrate embryos or tissues - Field research   Write "N/A" if the submission does not require an ethics statement.  General guidance is provided below. Consult the [submission guidelines](http://journals.plos.org/plosone/s/submission-guidelines#loc-guidelines-for-specific-study-types) for detailed instructions. Make sure that all information entered here is included in the Methods section of the manuscript. | Authorization was sought from the Jaramogi Oginga Odinga University of Science and  Technology, Board of Postgraduate studies. Ethical approval from Institutional  Scientific Ethics Review Committee of University of East Africa Baraton  (UEAB/ISERC/05/11/2024), thereafter research permit from the National Commission for Science, Technology and Innovations (NACOSTI P/24/42206) before the commencement of the study. Consent was obtained from all participants. It included purpose of the study, risk and benefits, voluntary participation, confidentiality and rights of the participants. To cater for the children after parents’ consent, assent was sought before proceeding to the survey. Data was anonymized and stored in accesscontrolled computers with access limited to the research team. No identifiable information was used in any study report, presentations or publications. |

Format for specific study types

Human Subject Research (involving human

participants and/or tissue)

Give the name of the institutional review

board or ethics committee that approved the

study

•

Include the approval number and/or a

statement indicating approval of this

research

•

Indicate the form of consent obtained

(

written/oral) or the reason that consent was

not obtained (e.g. the data were analyzed

anonymously)

•

Animal Research (involving vertebrate

animals, embryos or tissues)

Provide the name of the Institutional Animal

Care and Use Committee (IACUC) or other

relevant ethics board that reviewed the

study protocol, and indicate whether they

approved this research or granted a formal

waiver of ethical approval

•

Include an approval number if one was

obtained

•

If the study involved

non-human primates

,

add

additional details

about animal welfare

and steps taken to ameliorate suffering

•

If anesthesia, euthanasia, or any kind of

animal sacrifice is part of the study, include

briefly which substances and/or methods

were applied

•

Field Research

Include the following details if this study

involves the collection of plant, animal, or

other materials from a natural setting:

Field permit number

•

Name of the institution or relevant body that

granted permission

•

Data Availability

Authors are required to make all data

underlying the findings described fully

available, without restriction, and from the

time of publication. PLOS allows rare

exceptions to address legal and ethical

concerns. See the

[PLOS Data Polic](http://journals.plos.org/plosone/s/data-availability)

[y](http://journals.plos.org/plosone/s/data-availability)

and

[FA](http://journals.plos.org/plosone/s/data-availability#loc-faqs-for-data-policy)

[Q](http://journals.plos.org/plosone/s/data-availability#loc-faqs-for-data-policy)

for detailed information.

Yes - all data are fully available without restriction

| A Data Availability Statement describing where the data can be found is required at submission. Your answers to this question constitute the Data Availability Statement and will be published in the article, if accepted. |  |
| --- | --- |
| Important: Stating ‘data available on request from the author’ is not sufficient. If your data are only available upon request, select ‘No’ for the first question and explain your exceptional situation in the text box. |  |
| Do the authors confirm that all data underlying the findings described in their manuscript are fully available without restriction? |  |
| Describe where the data may be found in full sentences. If you are copying our sample text, replace any instances of XXX with the appropriate details.   - If the data are held or will be held in a public repository, include URLs, accession numbers or DOIs. If this information will only be available after acceptance, indicate this by ticking the box below. For example: All XXX files   are available from the XXX database (accession number(s) XXX, XXX.).   - If the data are all contained within the manuscript and/or Supporting Information files, enter the following: All relevant data are within the manuscript and its Supporting Information files. - If neither of these applies but you are able to provide details of access elsewhere, with or without limitations, please do so. For example:   Data cannot be shared publicly because of [XXX]. Data are available from the XXX Institutional Data Access / Ethics Committee (contact via XXX) for researchers who meet the criteria for access to confidential data.  The data underlying the results presented in the study are available from (include the name of the third party | All relevant data are within the manuscript |

| and contact information or URL).  • This text is appropriate if the data are owned by a third party and authors do not have permission to share the data.  * typeset |  |
| --- | --- |
| Additional data availability information: | Tick here if your circumstances are not covered by the questions above and you need the journal’s help to make your data available. |

Manuscript	[Click here to](https://www2.cloud.editorialmanager.com/pone/download.aspx?id=42437294&guid=b525e874-56b8-4f59-8f29-5165f8267762&scheme=1)

[access/download;Manuscript;Manuscriptv1_Lilian.docx](https://www2.cloud.editorialmanager.com/pone/download.aspx?id=42437294&guid=b525e874-56b8-4f59-8f29-5165f8267762&scheme=1)

1 **Factors Contributing to Low Tuberculosis Diagnosis Among Children Aged 0-14 Years in** 2 **Gem Sub County in Siaya County, Kenya.**

3 Lilian Atieno Okumu1*, Daniel Ogungu Onguru1, and David Otieno Odongo2 James Onyuro
4 Oketch1
5 1. School of Health Sciences, Jaramogi Oginga Odinga University of Science and Technology,
6 P.O. Box 210-40601, Bondo, Kenya
7 2. In-depth Research Services, Institute of Health and Social Work, Nairobi
8 ***Corresponding Author:** Lilian Otieno Okumu, Email: okumulilian@yahoo.com

# 9 ABSTRACT

10 Tuberculosis (TB) is a top global health challenge, with 1.2 million children aged 0–14 years
11 becoming ill with TB every year. Yet, a significant proportion remain undiagnosed or missed due
12 to diagnostic barriers. This cross-sectional mixed methods study investigated factors contributing
13 to low tuberculosis diagnosis among children in Gem Sub County, Siaya County Kenya, a high
14 burden region. Data was collected from 71 healthcare workers (HCWs) and 16 respondents across
15 six wards using semi-structured questionnaires, key informant interviews, and TB register
16 abstraction. Qualitative data underwent deductive thematic analysis while quantitative data was
17 analyzed using descriptive statistics and logistic regression in SPSS version 27. About a third
18 (31.3%) of TB cases in children required three or more facility visits before diagnosis, with some
19 needing up to 12 visits. Costly and inaccessible chest X-rays and GeneXpert underutilization due 20 to difficulties in sputum and alternative sample (gastric lavage, nasopharyngeal aspirates) 21 collection were key diagnostic constraints. Delayed care-seeking due to stigma, misconceptions 22 (56.3% linked childhood TB to HIV) and low TB symptom awareness (37.5% of children

23	presented with ≤2 symptoms) were patient related factors associated with low TB diagnosis. About

56.3% of caregivers sought routine TB screening, but 62.5% sought care only after persistent symptoms. Clinical officers were more confident in sample collection (OR=34, 95% CI 3.81– 303.21, p=0.002) and GeneXpert interpretation (OR=15, 95% CI 3.20–70.39, p<0.001) than nurses. Trained HCWs were 7.16 times more likely to interpret X-rays accurately (95% CI 2.16– 23.67, p<0.001). Enhanced HCW training, improved diagnostic access, targeted community education on stigma and misconceptions are critical for early TB detection in children.

**Key words:** Tuberculosis, diagnosis, Paediatrics, Siaya, Kenya

# INTRODUCTION

Tuberculosis (TB), caused by *Mycobacterium Tuberculosis*, transmitted through respiratory droplets from infected individuals, is a formidable global health challenge. In 2023, an estimated 10.8 million new cases occurred worldwide, including 1.3 million among children aged 0–14 years, equivalent to 12% of the total TB burden (1). Of these, 8.2 million people were reported as newly diagnosed, up from 7.5 million in 2022 and 7.1 million in 2019 (1). Furthermore, despite being preventable and treatable (2), TB causes deaths, claiming approximately 1.3 million lives annually (1). Yet, many cases remain undiagnosed or underreported (3). In particular, pediatric population. This is mainly attributed to nonspecific symptoms, low bacillary loads, and challenges in sputum sample collection in children, and limited access to diagnostic tools in high-burden settings (4, 5).

Kenya, ranking fourth in Africa for TB burden after Ethiopia, Nigeria, and South Africa, is among the top 10 high-burden countries globally. The Kenya National TB prevalence survey of 2015/2016, reported a TB prevalence of 558 per 100,000 population with nearly half of the cases missed annually (6). Notably, 21% of the patients with TB symptoms sought prior care at private clinics and chemists (6). TB is often under-reported, with children representing only 8% of the 2,903 TB cases reported in Siaya County in 2021 according to Kenya TB surveillance data 2021, below the national target of 12%. In Gem Sub-County, only 4% of the 619 TB cases reported in 2021 were pediatric, well below the expected 10–15% range, highlighting significant diagnostic gaps.

Multiple factors drive TB under-diagnosis in pediatric population. This includes the absence of clear clinical features, diagnostic tool limitations, and patient- and facility-related barriers (7, 8). A systematic review by (7) demonstrated that TB symptom-based screening is inconsistently implemented. Patients attending health facilities are not consistently asked about respiratory symptoms nor promptly offered diagnostic tests once the symptoms are identified. Under-reporting and misdiagnosis contribute to delayed treatment, ongoing community transmission and risk of developing drug-resistant TB. Hence, the accurate and timely identification of TB in pediatric populations is critical for effective treatment and preventing community transmission.

Despite these challenges, local data on pediatric TB diagnosis barriers remain limited. This is concerning given the burden of TB in Kenya. The reliability of GeneXpert in children, limited by low bacillary loads, remains under scrutiny, necessitating further investigation, to address the rising burden. This study investigated the constraints to diagnostic approaches, patient-related, and facility-based barriers to pediatric TB diagnosis in Gem Sub-County, Siaya County. By identifying these barriers, the findings aim to inform interventions to improve early detection, reduce missed cases, and support TB control efforts in Siaya County and similar geographic areas.

**METHODOLOGY**

# Study Area and Design

This cross-sectional study was conducted in Gem sub-county, Siaya County, Western Kenya with participants recruited between 13^th^ December 2024 and 16^th^ January 2025. Siaya County has one of the 10 highest burden TB Counties in Kenya. The county consists of six sub-counties. The study specifically focused on Gem sub-county, which has the highest tuberculosis notification rate, as reported in the National tuberculosis, leprosy, and lung disease 2022 annual report. Gem subcounty is served by four levels of healthcare facilities: Level 1 (community health services), Level 2 (dispensaries), Level 3 (health centers), and Level 4 (sub-county hospitals).

# Study Population

The study population comprised children aged 0–14 years diagnosed with TB at health facilities in Gem Siaya, and receiving treatment during the study period, provided they or their caregivers gave written informed consent or assent. Healthcare workers (HCWs), including doctors, nurses, clinical officers, laboratory technologists, and pharmaceutical technologists, employed for at least two months with pediatric TB experience and consented were included. Exclusions included

HCWs with less than two months’ employment, or lacking pediatric TB experience, and children transferred from other facilities.

# Sampling Technique

A multistage sampling approach was used. Twenty TB diagnostic facilities (two sub-county hospitals, health centers, dispensaries, and private/mission facilities) were purposively selected based on high pediatric TB caseloads. Pediatric TB cases were abstracted from the TIBU register, with 16 patients/caregivers selected via simple random sampling. Stratified sampling ensured representation across HCW cadres, with 71 HCWs purposively selected for pediatric TB experience.

# Sample Size Calculation

Sample size was determined using the Taro Yamane formula (Yamane, 1967), yielding 76 HCWs and 16 patients/caregivers.

The formula is defined as:

104 where:
105 n = desired sample size
106 N = population size
107 e = acceptable margin of error
108 For the number of caregivers/patients, the sample size is calculated as:

110 For the HCWs, the sample size is calculated as;
111

112

This implied that approximately 76 HCWs and 16 patients' /caregivers or guardians were sampled from the selected health facilities to achieve a 95% confidence level with a 5% margin of error, assuming a response distribution of 50%.

# Data Collection Procedure

Trained research assistants used the Kobo Collect platform on Android devices to administer semistructured questionnaires, capturing HCW knowledge of TB, diagnostic practices, and challenges encountered in diagnosing TB in children. Open-ended interviews with 16 caregivers/children explored sociodemographic data, healthcare-seeking behaviors, and barriers. TB4 register abstraction provided case data in selected facilities. Consent forms were provided in English and local languages prior to participation, with assent obtained for children. Tools were pretested to ensure reliability, and daily debriefings with assistants ensured data quality.

# Data Management and Analysis

Data was extracted to Excel, cleaned, coded, and imported into SPSS version 27 for analysis. Descriptive statistics was used to summarize characteristics of HCWs, pediatric TB cases, and diagnostic constraints and practices. Bivariate analysis was used to determine associations between independent variables (HCW training, cadre) and specific outcomes related to low TB diagnosis; (1) accurate chest X-ray interpretation (defined as correctly identifying TB-related abnormalities per WHO guidelines), (2) confidence in sample collection (self-reported ability to collect sputum or alternative samples), and (3) confidence in GeneXpert interpretation (self-reported ability to interpret GeneXpert results). Univariate binary logistic regression was used to calculate odds ratios (ORs) for each predictor-outcome pair separately to explore associations, with a p-value < 0.05 considered statistically significant. Due to multiple distinct outcomes and a small sample size (71 HCWs, 16 patients/caregivers), multivariate analysis was not performed to avoid overfitting, and no correction for multiple comparisons was applied given the exploratory nature of the study. Qualitative data from interviews were transcribed, de-identified, and analyzed via deductive thematic analysis, with open and axial coding to identify themes.

# Ethical Considerations

Authorization was sought from the Jaramogi Oginga Odinga University of Science and

Technology, Board of Postgraduate studies. Ethical approval from Institutional Scientific Ethics Review Committee of University of East Africa Baraton (UEAB/ISERC/05/11/2024), thereafter research permit from the National Commission for Science, Technology and Innovations (NACOSTI P/24/42206) before the commencement of the study. Consent was obtained from all participants. It included purpose of the study, risk and benefits, voluntary participation, confidentiality and rights of the participants. To cater for the children after parents’ consent, assent was sought before proceeding to the survey. Data was anonymized and stored in access-controlled computers with access limited to the research team. No identifiable information was used in any study report, presentations or publications.

**RESULTS**

# Sociodemographic Characteristics of the Respondents

The study involved 71 healthcare workers and 16 respondents (13 parents/guardians and 3 children). About half (38, 53.5%) were female. Similarly, over half (9, 56.3%) were aged 35 years and above. Majority (44, 62%) were aged 21-34 years. Most HCWs (55, 77.5%) had up to diploma training, 11(15.5%) had a degree, and a smaller proportion (5, 7%) had only secondary education.

Table 4.1

# Table 4.1: Sociodemographic Characteristics of the Respondents

**Healthcare workers**

**Variable**

**Category**

**Frequency (n=71)**

**Percentage**

| Gender | Female | 38 | 53.5 |
| --- | --- | --- | --- |
|  | Male | 33 | 46.5 |
| Age Category | 21-34 years | 44 | 38 |
|  | 35+ years | 27 | 62 |
| Education level | Secondary | 5 | 7 |
|  | Diploma | 55 | 77.5 |
|  | Degree | 11 | 15.5 |
| Cadre | Clinical Officer | 18 | 25.4 |
|  | Nurse | 24 | 33.8 |
|  | Laboratory Technologist | 15 | 21.1 |
|  | Peer educator | 4 | 5.6 |
|  | AYP | 3 | 4.2 |
|  | HTS provider | 2 | 2.8 |
|  | Nutritionist | 2 | 2.8 |

Pharmaceutical Technologist	1	1.4

Link assistant	1	1.4

TB champion	1	1.4

SCTLC	1	1.4

Work Experience	< 2 years	13	18.3

3-5 years	16	22.5

6-9 years	21	29.6

10+ years	21	29.6

Work duration in 5+ years	16	22.5

current facility	1-4 years	55	77.5

Facility	Dispensary	7	9.9

Health Centre	48	67.6

Sub-county Hospital	16	22.5

Current Employer	MOH	34	47.9

MOH/Partner support	20	28.2

Faith based	7	9.9

Private	10	14.1

**Clients**

Variable	Category	Frequency (n=16)	Percentage

Person interviewed	Client	3	18.8

Parent/guardian	13	81.3

Gender	Female	13	81.3

Male	3	18.8

Age in years	12-18	3	18.8

19-34	4	25

35+	9	56.3

Education level	Primary	10	62.5

Secondary	4	25

College	2	12.5

**Diagnostic Constraints for Paediatric Tuberculosis**

# Chest X-ray Constraints

High costs and limited access to chest X-ray facilities were reported barriers. A 34-year-old nurse stated, *“Most patients cannot afford X-ray charges, hindering proper diagnosis.”* A 29-year-old clinical officer pointed out, *“one would use over 2000/= just to get the X-ray done, while at home they lack food.”* Only 23.9% of HCWs could interpret pediatric radiographs, citing poor image quality and inadequate training. A 40-year-old laboratory technologist noted, *“X-ray interpretation requires highly trained personnel, often unavailable in remote settings.”* Additionally, a 27-year-old CO warned, *“wrong positioning of the patient could lead to wrong interpretation and hence incorrect diagnosis.”*

Radiation concerns and transportation costs further limited use, with 41.2% of imaging completed within 12 hours. A 33-year-old link assistant noted, *“Chest X-rays are not advisable in children due to harmful rays, which may lead to missed cases due to fear.”* Additionally, a 40-year-old CO reported, *“Facilities equipped with X-ray equipment are quite a distant from patients reach.”* making referrals a challenge.

# Gene X-pert/Smear Microscopy Challenges

Sample collection challenges were reported in young children unable to expectorate sputum. Alternative methods (gastric lavage, nasopharyngeal aspirates) required specialized skills, lacking in many HCWs. Stock-outs of GeneXpert cartridges, nasogastric tubes, and reagents disrupted diagnosis. A 26-year-old link assistant noted, "*Obtaining samples from infants and pediatrics who are not able to expectorate is a challenge due to inadequate trained personnel in nasopharyngeal aspiration*.” A 45-year-old CO noted, “*Stock-outs of NG tubes and essential supplies disrupt sample collection.”* Additionally, a 32-year-old CO noted, *"Transportation of the samples from facility A to the laboratory hubs interferes with sample quality,”* while a 40-year-old Nursing officer emphasized high costs. *"It is expensive to transport the sample to a definitive facility."*

# WHO Diagnostic Algorithm Constraints

Underuse of TB algorithm was attributed to complexity and lack of training (69% of HCWs lacked recent pediatric TB training). Algorithms were not available at all service delivery points, and frequent updates without dissemination led to misinterpretation. A 48-year-old nurse reported,

*“Misunderstanding of algorithms requires regular sensitizations.”* A 29-year-old CO noted, *"Algorithms keep changing especially for management of pediatrics on TB treatment."* A 44-yearold clinician stated, *"Not all service delivery points (SDPs) have access to the algorithms.”* (Table 4.2)

206

207

208

209	**Table 4.2: Summary of Key Insights on Constraints to TB Diagnosis.**

| **Diagnostic**  **Approach** | **Emerging**  **Themes** | **Key Insights** | **Representative Quotes** |
| --- | --- | --- | --- |
| Chest X-  Ray | Cost and  Accessibility  Constraints | High costs and distance to facilities hinder early detection | “One would use over 2000/= just to get the X-ray done, while at home they lack food.” (29-year-old CO) |
|  | Image Quality &  Interpretation  Issues | Poor image quality and lack of trained personnel lead to misdiagnosis. | “Wrong positioning of the patient could lead to wrong interpretation and hence incorrect diagnosis.” (27year-old CO) |
| GeneXpert/  Smear  Microscopy | Sample  Collection  Difficulties | Young children cannot expectorate sputum,  making sample collection  difficult. | “Gastric aspirate collection in under-fives is not easy as it requires much courage.” (Nursing Officer) |
| WHO  Diagnostic  Algorithms | Lack of Training and Awareness on Updates | Frequent updates without dissemination result in misinterpretation and poor implementation. | “Misunderstanding of the algorithms hence need for regular sensitizations.” (48-year-old Nurse) |

Misinterpretation	Some healthcare workers “Misinterpretations are common.”

and Complexity find the algorithms difficult (30-year-old Laboratory of Algorithms to interpret and apply. Technologist)

# Patient Related Factors Contributing to Low Tuberculosis Diagnosis in Children

Delayed care-seeking was common due to low symptom awareness (37.5% of children had ≤2 symptoms) A 62-year-old woman admitted, *"I did not know the signs and symptoms of TB let alone in children,"* A 27-year-old woman initially dismissed symptoms in a child because she believed TB only affected older people. *"I assumed TB was a disease of the old,"* she confessed. Further, financial constraints and long distances to facilities were barriers. A 32-year-old woman expressed her struggles: *"No money and long distance in accessing health service makes it difficult."*

Similarly, a 38-year-old and a 14-year-old girl struggled with reaching healthcare facilities in time.

Stigma and misconceptions (56.3% linked TB to HIV, 37.5% believed TB was hereditary or a curse) delayed diagnosis, with fears of community judgment. A 49-year-old woman hesitated taking her child for TB diagnosis due to stigma. She admitted, *" Stigma of being diagnosed with TB. Fear of the community’s perception of those who have TB could be having HIV/AIDs delayed my decision."* A 37-year-old mother couldn’t believe a young child could have TB. *"I didn’t think such a young child could have TB."* For a 12-year-old girl, the possibility of TB was only considered only after further testing. *"Nobody thought it was TB until the doctor suggested I go for an X-ray. It could be TB,"* she recalled

Quantitative findings confirmed these. Over half (56.3%) of caregivers sought routine TB screening, but 62.5% only sought care after persistent symptoms. Most respondents (14, 87.5%) knew at least three TB signs and symptoms. Over a third (6,37.5%) of children presented with only

0–2 symptoms before diagnosis. Notably, slightly over half (9,56.3%) associated TB with HIV. (Table 4.3). Most children (75%) presented with cough, followed by weight loss (62.5%) and chest pain (56.3%) (Figure 4.4). No significant differences in facility visits were found by gender (p=0.1495) or symptom knowledge (p=0.7055) (Table 4.4).

12.5

12.5

31.3

6.3

18.8

6.3

6.3

6.3

0

5

10

15

20

25

30

35

1

2

3

4

5

6

7

12

**Percentage diagnosed with TB**

**Number of Visits**

**Figure 4.2: Number of Health Facility Visits before TB diagnosis**

# Table 4.3 Patient Related Factors Contributing to Delayed TB Diagnosis

**Variable**

**Fre**

**quency**

**Percentage**

# Know signs and symptoms of TB

No	2	12.5

Yes	14	87.5

# Number of TB known signs and symptoms

| 0 2 | 12.5 |
| --- | --- |
| 3+ 14  **Number of signs and symptoms child presented with before diagnosis** | 87.5 |
| 0-2 6 | 37.5 |
| 3+ 10  **Myths about Tuberculosis disease** | 62.5 |
| TB is a curse 6 | 37.5 |
| TB is not curable 2 | 12.5 |
| TB is hereditary 6 | 37.5 |
| If you have TB then you have HIV 9 | 56.3 |
| TB is for people who drink alcohol 1  **Take children under your care to hospital for TB screening** | 6.3 |
| No 7 | 43.8 |
| Yes 9  **Point at which you take children to hospital** | 56.3 |
| To perform early diagnosis of TB 4 | 44.4 |
| Routine check for TB disease 3  Do not undergo early screening until signs and | 33.3 |
| symptoms manifest 2  **Where children are taken for TB screening** | 22.2 |
| Public hospital 9 | 69.2 |
| Private hospital 3 | 23.1 |

| Community level (CHP)  **Where I seek help when a child falls sick** | 1 | 7.7 |
| --- | --- | --- |
| Buy medicine at the chemist/pharmacy | 4 | 25 |
| Go to health facility | 12 | 75 |

# Point at which child is taken to health facility after onset of illness

Immediately a child falls sick	6	37.5

When symptoms persist	10	62.5

Cough of any duration was the most common symptom (87.5%), and most children (75%) presented with it prior to diagnosis. Chest pain was identified by 87.5% of respondents but was present in only 56.3% of children before diagnosis. Weight loss was acknowledged by 87.5% of respondents, but only 62.5% of children presented with it. Fever was mentioned by 62.5% of respondents, yet only 37.5% of children experienced it before diagnosis. Night sweats were identified by 56.3% of respondents but present in only 43.8% of children. (Figure 4.4)

TB signs and symptoms mentioned by Respondents

TB signs and symptoms child presented with just before diagnosis

**Proportion(%)**

| 87.5 |  | 87.5 |  | 87.5 |  |  |  |
| --- | --- | --- | --- | --- | --- | --- | --- |
|  | 75 |  | 56.3 | 62.5 | 62.5 |  | 56.3 |
|  |  |  |  |  |  | 37.5 | 43.8 |

**Cough of any	Chest pain	Weight loss	Fever	Night sweats duration**

**TB signs and symptoms**

251

# 252 Figure 4.4: TB signs and symptoms mentioned and those children presented with

253

254 Table 4.4 shows there was no significant difference in facility visit for TB diagnosis based on 255 gender (p = 0.1495), knowledge of TB symptoms (p = 0.7055), and number of known TB signs &

256	symptoms (p = 0.7055).

# 257 Table 4.4: Facility Visits by Gender and Knowledge of TB Signs and Symptoms

| **Variable** | **Group 1 (n)** | **Rank**  **Sum 1** | **Group 2**  **(n)** | **Rank z-value**  **Sum 2** | **p-value** |
| --- | --- | --- | --- | --- | --- |

Gender

Female (13)

101.5

Male (3)

34.5

-

1.441

0.149

| Knowledge of TB Signs &  Symptoms | No (2) | 15 | Yes (14) | 121 | -0.378 | 0.705 |
| --- | --- | --- | --- | --- | --- | --- |
| Number of Known TB Signs &  Symptoms | 0 (2) | 15 | ≥3 (14) | 121 | -0.378 | 0.705 |

258

# Facility Related Factors Influencing Tuberculosis Diagnosis in Children

Only 31% of HCWs had recent pediatric TB training, with 26 (53.1%) citing lack of opportunity or unawareness of such training (12, 24.5%). Most HCWs (65, 91.5%) could correctly identify three or more signs of active TB, but only 17(23.9%) could interpret pediatric radiographs. Most respondents (48, 67.6%) believed that all HCWs, regardless of cadre, should receive training on childhood TB management. Almost all respondents (69, 97.2%) were aware that a child can be infected with TB more than once in their lifetime. Table 4.5

# Table 4.5: Healthcare Worker Related Factors on Childhood TB Diagnosis

**Variable**

**Frequency**

**Percentage**

# Attended training /workshop on Pediatric TB in the past 12 months

| No | 49 | 69 |
| --- | --- | --- |
| Yes  **If No Why** | 22 | 31 |
| Was committed to other duties | 9 | 18.4 |
| Not aware that the above training exists | 12 | 24.5 |
| Not invited/provided an opportunity | 26 | 53.1 |
| There has been no training specific to pediatric TB  **Who should be trained on childhood TB Management** | 2 | 4.1 |
| Specific group of HCWs | 23 | 32.4 |
| All HCWs regardless of cadre | 48 | 67.6 |

# Number of TB Signs and symptoms mentioned by Respondents

0-2 signs	6	8.5

3+ signs	65	91.5

**A child can be infected with TB more than once in their lifetime**

Don’t know	2	2.8

Yes	69	97.2

**Situations when Patients are given health education messages about TB**

During World TB Day	61	86

During BCG Immunization	55	77.4

During General health promotion/ education delivered in clinical

settings	69	97.1

With suspected or confirmed cases only (i.e. no family members)	5	70.4

With suspected cases and their families in a clinical setting	58	81.7

With confirmed patients and their families	60	84.5

Health education on TB is generally not done with patients	1	1.4

**Primary diagnostic test to confirm or rule out active TB in children**

GeneXpert	35	49.3

Chest X Ray	10	14.1

Sputum smear microscopy	15	21.2

TB LAM	11	15.5

**Ever collected sample for TB diagnosis**

No	30	42.3

Yes	41	57.7

# If yes type of sample collected

Sputum	17	41.5

Gastric aspirate	15	36.6

Nasopharyngeal aspirate	9	22

**How confident are you in collecting sample from Children**

Very confident	16	22.5

Confident enough	26	36.6

Not so sure	29	40.8

**Confidence in interpreting microscopy, GeneXpert TB results in children**

No	32	45.1

Yes	39	54.9

**Able to interpret radiography images in children**

No	54	76.1

Yes	17	23.9

**Ever diagnosed TB disease in a child**

No	34	47.9

Yes	37	52.1

**Method used to diagnose TB in a child**

Clinical diagnosis (algorithm guide by WHO)	24	33.8

GeneXpert test	20	28.2

Smear microscopy	8	11.3

Other	1	1.4

# Skill/ training level needed to effectively conduct DOT in TB patient

Any healthcare worker regardless of clinical training	33	46.5

Healthcare worker with clinical training	22	31

This is not a technical activity that requires training	15	21.1

Only highly qualified trained healthcare workers	1	1.4

Multiple facility visits were required before diagnosis as noted by a 42-year-old parent, *“We sought help from different big hospitals within the County but in vain, the 6th visit is what came to our rescue”* A 30-year-old woman echoed this saying, *"The medics failed to get the right diagnosis on time."* Similarly, a 12-year-old girl was only diagnosed after further testing. *"Nobody thought it was TB until the doctor suggested I go for an X-ray. It could be TB."* A 51-year-old man expressed frustration after numerous visits without receiving proper guidance. *"There was no proper medication all this time. I was visiting different facilities with my child. No one ever mentioned to me about required tests."*

Most respondents were managed for other conditions during more than one visit for TB diagnosis. (Figure 4.3). This was confirmed by HCWs noting that TB symptoms in children are not easily identifiable due to similarity with other diseases. *“Most children diagnosed don’t present with easily identifiable symptoms,”* noted a 48-year-old Nurse. A 27-year-old Laboratory Technologist reiterated, *“The similarity of TB signs and symptoms to other diseases makes clinical diagnosis hard.* “Additionally, the HCWs identified persistent shortage of adequately trained personnel. A

40-year-old registered nurse stated, *"Lack of enough trained personnel, and understaffing make*

*TB diagnosis difficult."*

**Sent for chest X-ray… Sent for malaria test…**

**Several tests done**

**7%Medication**

**Sent for sputum test_…_^65%^**

# Figure 4.3: Management Plans for Children with TB Symptoms at health facilities

Clinical officers were more confident in sample collection (OR 34, 95% CI 3.81–303.21, p=0.002) and GeneXpert interpretation (OR 15, 95% CI 3.20–70.39, p<0.001) than nurses (Table 4.6). HCWs with training were 7.16 times more likely to interpret chest X-rays (95% CI 2.16–23.67, p<0.001) and 7.15 times more confident in sample collection (95% CI 1.87–23.36, p=0.004) (Table 4.7).

# Table 4.6: Facility/Healthcare worker Related Factors on Childhood TB Diagnosis

Variable

OR

95

% CI

P value

# Confidence collecting samples from children

| Nursing Officer | Ref |  |  |
| --- | --- | --- | --- |
| Clinical Officer | 34 | (3.81-303.21) | 0.002 |
| Laboratory Technologist | 4 | (1.01-15.71) | 0.047 |

# Confidence Interpreting Microscopy/ GeneXpert MTB/R results

| Nursing Officer | Ref |  |  |
| --- | --- | --- | --- |
| Clinical Officer | 15 | (3.196-70.392) | <0.001 |

| Laboratory Technologist 1 (empty)  **Ever collected sample for TB diagnosis in children** |  |
| --- | --- |
| Nursing Officer Ref |  |
| Clinical Officer 34 (3.812-303.215) | 0.002 |
| Laboratory Technologist 4 (1.017-15.717)  **Major signs and symptoms of active TB disease** | 0.047 |
| Nursing Officer Ref |  |
| Clinical Officer 0.73 (0.043-12.673) | 0.835 |
| Laboratory Technologist 0.17 (0.016-1.857)  **Attended training workshop for TB diagnosis in the past 12 months** | 0.148 |
| Nursing Officer Ref |  |
| Clinical Officer 10 (2.337-42.782) | 0.002 |
| Laboratory Technologist 0.35 (0.035-3.545)  **Able to interpret chest radiography images in Children** | 0.379 |
| Nursing Officer Ref |  |
| Clinical Officer 55 (8.179-369.848) | <0.0001 |
| Laboratory Technologist 1 (empty)  **Who should be trained on childhood TB diagnosis and Management** |  |
| Nursing Officer Ref |  |
| Clinical Officer 0.52 (0.139-1.967) | 0.338 |
| Laboratory Technologist 0.91 (0.210-3.989)  **Ever diagnosed TB disease in a child** | 0.908 |
| Nursing Officer Ref |  |

Clinical Officer	7	(1.590-30.799)	0.01

Laboratory Technologist	1.6	(0.436-5.865)	0.478

# Table 4.7: Training Workshop Attendance on Chest X-ray Interpretation/Sample Collection

| **Variables in the equation B S.E.** | | **Wald** | **df** | **Sig.** | **Exp(B)** | **95% C.I.for**  **EXP(B)**  **Lower** | **Upper** |
| --- | --- | --- | --- | --- | --- | --- | --- |
| **Confidence on Chest X Ray Interpretation** |  |  |  |  |  |  |  |
| Step training_wkshop_peds_  1a TB_past_12months (1) 1.969 | 0.610 | 10.434 | 1 | 0.001 | 7.167 | 2.169 | 23.675 |
| Constant -1.969 | 0.436 | 20.423 | 1 | <0.0001 | 0.139 |  |  |
| **Confidence in Sample Collection**  Step training_wkshop_peds_  1a TB_past_12months (1) 1.968 | 0.684 | 8.281 | 1 | 0.004 | 7.159 | 1.873 | 27.360 |
| Constant -0.123 0.286 0.183 1 0.668    a. Variable(s) entered on step 1: training_wkshop_peds_TB_past_12months. | | | | | 0.884 |  |  |

**DISCUSSION**

# Introduction

The study identified multiple constraints to Pediatric TB diagnosis within Gem Sub County. Diagnostic constraints included costly and inaccessible chest X-rays, GeneXpert underutilization due to sample collection challenges, and underuse of WHO algorithms due to complexity and inadequate training (69% of HCWs lacked recent training). Delayed care-seeking due to low symptom awareness, stigma (56.3% linked TB to HIV), and misconceptions, contributed to diagnostic delays. Approximately 31.3% of cases required ≥3 facility visits, with some needing up to 12. Facility-level issues included HCW knowledge gaps, understaffing, and shortages of diagnostic tools (e.g., GeneXpert machines, reagents). Herein we discuss these findings.

# Constraints to Diagnostic Approaches for Tuberculosis in Children

High costs and limited access to chest X-rays align with findings from resource-constrained settings, where financial barriers including transportation costs hinder timely TB diagnosis (9). TB patients in resource-constrained settings, often incur unmanageable costs while seeking and staying in care for the full duration of anti-TB treatment(9). Poor image quality and low HCW training (only 23.9% could interpret Paediatric radiographs) contribute to diagnostic inaccuracy. These findings complement (5), who indicated current diagnosis as suboptimal due to challenges in sample collection, and inadequate access to diagnostic tools in healthcare settings in high burden areas. In Ivory coast, Cameroon, Cambodia, Uganda among other countries (10) access to radiography significantly improved TB detection rates in children.

Pediatric sample collection difficulties for GeneXpert, reflect WHO’s note on practical challenges with nasopharyngeal aspirates and gastric lavage. These specimens (gastric lavage and nasopharyngeal aspirates) have highest bacterial yield. However, they require specialized skills in collection except for stool sample collection. Yet, this study revealed that many healthcare workers lacked the required skills to collect them. Healthcare workers reported that TB symptoms in children are not easily identifiable leading to many cases remaining misdiagnosed, not-diagnosed, or untreated. This is consistent with findings from other studies highlighting the non-specific nature of TB symptoms in children as a major diagnostic challenge (WHO, 2022).

Frequent stock-outs of GeneXpert cartridges and reagents echo global reports of supply chain disruptions in low-resource settings. Underuse of WHO algorithms due to complexity and inadequate training corroborates with a study in Zambia implementation of multicomponent strategy led to high case notifications in children (11). (12), emphasizes the need for simplified, disseminated algorithms to address the poor integration of TB screening into pediatric care. Taken together, diagnosis of TB in children relies on thorough assessment of all the evidence derived from a careful history of exposure, clinical examination and relevant investigations. Although bacteriological confirmation of TB is not always feasible, it should be sought whenever possible by microscopy, culture or WHO-endorsed genotypic (molecular) testing (i.e., X-pert MTB/RIF) of respiratory or non-respiratory samples to provide diagnostic support.

# Patient-related Factors Affecting Tuberculosis Diagnosis in Children

Many caregivers and even older children failed to recognize TB symptoms, hence delaying their decision to seek medical attention. The low symptom awareness and misconceptions (e.g., 56.3% linked TB to HIV) aligns with the 2016 Kenya TB Prevalence Survey, where 64.9% of symptomatic cases had not sought any health care prior to the survey (6). A systematic review by (7) demonstrated inefficiencies in TB screening based on symptoms as a main contributor to underdiagnosis. Those presenting with TB symptoms and offered a diagnostic investigation was very low with a study level median of 38% (IQR 14–44, range4–84). Reactive approach to healthcare disadvantages TB detection and treatment, leading to significant underreported or missed cases. The WHO Report 2021, posits that TB detection in children is particularly disadvantaged due to the paucibacillary nature of pediatric TB, the overlap of nonspecific symptoms with other childhood illnesses, and that most children do not excrete enough bacilli to be detectable by available bacteriological tests (WHO Report 2021, Module 5). These barriers impede early TB detection and treatment initiation.

Misconceptions that TB primarily affects adults, stigma, contributed to symptom neglect in pediatric cases. Stigma, particularly TB’s association with HIV, is a well-documented barrier to timely diagnosis across high-burden settings. Reference? The high number of facility visits (up to 12) before diagnosis reflects missed opportunities in childhood TB diagnosis as seen in a scoping review by (13) and Nigeria, where non-adherence to guidelines led to missed cases (14). Improved training and mentorship of healthcare providers on standardized diagnostic protocols may improve TB detection in pediatric population.

**Facility/ healthcare worker Factors Affecting Tuberculosis Diagnosis in Children.**

The study revealed low training uptake (31% of HCWs trained) and cadre differences in diagnostic confidence (e.g., clinical officers being more confident). Reasons cited ranged from lack of invitations or being provided a chance to being unaware of training opportunities. Non-awareness suggests gaps in information dissemination rather than a lack of willingness to participate in training. Consistent with this study, (15) emphasized that structured training programs significantly improve TB detection rates by enhancing HCWs' knowledge and diagnostic confidence. Similarly, a study in Nigeria found that undertrained HCWs were less likely to adhere to national TB diagnostic protocols, leading to prolonged case identification (14). Research in Cameroon (16) found that HCWs with more extensive medical training, such as general practitioners, demonstrated better TB diagnostic skills due to their participation in workshops and professional exchanges. Targeted training programs that address the specific needs of different HCW groups are needed.

Shortages of diagnostic tools and stock-outs are consistent with global challenges in TB case detection (WHO, 2021). Given that Gene X- pert is the preferred diagnostic test for childhood TB, it is concerning that only 49.3% of HCWs identified it as the primary test. Additionally, difficulty in obtaining quality sputum samples from children disadvantaged TB diagnosis, necessitating alternative diagnostic methods such as gastric aspirates. Yet, a few HCWs had confidence in collecting alternative samples, in particular gastric lavage. The absence of essential tools, coupled with poor sample collection techniques, was a significant constraint to TB diagnosis. According to (12) for children with TB symptoms in settings without resources, an algorithmic approach may guide the initiation of TB treatment initiation, when imaging or microbiological testing are out of reach. Our findings confirm that diagnostic delays in pediatric TB also stem from systemic deficiencies in medical infrastructure and resource availability

The study had strengths and limitations. This study benefitted from a mixed methods approach effectively capturing the diagnostic challenges faced in childhood TB detection. The study included children or parents/guardians whose children had a confirmed TB disease. This prevented bias in the observed associations in relation to the true underlying association. However, reliance on self-reported data may have introduced biases. In particular, healthcare workers might have provided desirable responses regarding their TB diagnostic practices.

In conclusion, low pediatric TB diagnosis in Gem Sub-County stems from diagnostic

inaccessibility, patient delays due to stigma and awareness gaps, financial difficulties and facility /healthcare worker constraints (e.g., undertraining, resource shortages, need for multiple visits before diagnosis). Insufficient training of healthcare workers, a lack of confidence in interpreting diagnostic tests or collecting alternative pediatric specimens, and resource constraints impede TB diagnosis. Siaya County’s Ministry of Health should conduct refresher HCW training on pediatric TB diagnostics, facilitate sample referrals and X-ray services. Additionally, support community campaigns to reduce stigma and promote early screening. Future research should explore influence of TB integration into maternal child health programs on TB screening and detection in children in particular, high-burden settings.

**DATA AVAILABILITY.**

The data that support this study may be shared upon reasonable request to the corresponding author, if appropriate.

# CONFLICTS OF INTERESTS

The authors declare that they have no conflicts of interest.

**DECLARATION OF FUNDING.**

The study received no external funding

# AUTHOR CONTRIBUTIONS

LAO, DO, and DO conceived and designed the study. LAO drafted the manuscript. DO, DO and

JO reviewed the manuscript. All authors read and approved the final manuscript.

# ACKNOWLEDGEMENTS

We thank the study participants for their participation in the study. We appreciate the Ministry of Health County Government of Siaya for the permission to conduct this study. We are grateful to the management of health facilities in Siaya, Kenya, for their support during the study.

# REFERENCE

1. WHO. Global tuberculosis report 2024. Geneva: World Health Organization; 2024. Licence: CC BY-NC-SA 3.0 IGO.; 2024.
2. Bhargava A, Bhargava M. Tuberculosis deaths are predictable and preventable: Comprehensive assessment and clinical care is the key. J Clin Tuberc Other Mycobact Dis. 2020;19:100155.
3. Linn AR, Dubois MM, Steenhoff AP. Under-Reporting of Tuberculosis Disease among Children and Adolescents in Low and Middle-Income Countries: A Systematic Review. Trop Med Infect Dis. 2023;8(6).
4. Oliwa JN, Gathara D, Ogero M, van Hensbroek MB, English M, Van't Hoog A, et al. Diagnostic practices and estimated burden of tuberculosis among children admitted to 13 government hospitals in Kenya: An analysis of two years' routine clinical data. PLoS One. 2019;14(9):e0221145.
5. Basile FW, Nabeta P, Ruhwald M, Song R. Pediatric Tuberculosis Diagnostics: Present and Future. J Pediatric Infect Dis Soc. 2022;11(Supplement_3):S85-S93.
6. Enos M, Sitienei J, Ong'ang'o J, Mungai B, Kamene M, Wambugu J, et al. Kenya tuberculosis prevalence survey 2016: Challenges and opportunities of ending TB in Kenya. PLoS One. 2018;13(12):e0209098.
7. Divala TH, Lewis J, Bulterys MA, Lutje V, Corbett EL, Schumacher SG, et al. Missed opportunities for diagnosis and treatment in patients with TB symptoms: a systematic review. Public Health Action. 2022;12(1):10-7.
8. Zhou Y, Wang F, Huang L, Liu K, Zhang Y, Luo D, et al. Factors associated with tuberculosis care-seeking and diagnostic delays among childhood pulmonary tuberculosis in Zhejiang Province, China: a 10-year retrospective study. Sci Rep. 2024;14(1):17086.
9. Pedrazzoli D, Lalli M, Boccia D, Houben R, Kranzer K. Can tuberculosis patients in resource-constrained settings afford chest radiography? European Respiratory Journal. 2017;49(3).
10. Melingui BF, Basant J, Taguebue JV, Massom DM, Leroy Terquem E, Norval PY, et al. Implementation of digital chest radiography for childhood tuberculosis diagnosis at district hospital level in six high tuberculosis burden and resources limited countries. Trop Med Int Health. 2024;29(11):979-89.
11. Kagujje M, Nyangu S, Maimbolwa MM, Shuma B, Mutti L, Somwe P, et al. Strategies to increase childhood tuberculosis case detection at the primary health care level: Lessons from an active case finding study in Zambia. PLoS One. 2023;18(7):e0288643.
12. Gunasekera KS, Vonasek B, Oliwa J, Triasih R, Lancioni C, Graham SM, et al. Diagnostic Challenges in Childhood Pulmonary Tuberculosis-Optimizing the Clinical Approach. Pathogens. 2022;11(4).
13. Russo DO, Jimenez ALL, Diniz LMO, Cardoso CA, Romanelli RMC. Missed opportunities in the prevention and diagnosis of pediatric tuberculosis: a scoping review. J Pediatr (Rio J). 2024;100(4):343-9.
14. Ukwaja K, Alobu I, Onu E. Frontline healthcare workers' knowledge of tuberculosis in rural south-east Nigeria. African Journal of Respiratory Medicine. 2013;9(1).
15. Wu P-S, Chou P, Chang N-T, Sun W-J, Kuo H-S. Assessment of changes in knowledge and stigmatization following tuberculosis training workshops in Taiwan. Journal of the Formosan Medical Association. 2009;108(5):377-85.
16. Vukugah TA, Akoku DA, Tchoupa MM, Lambert E. Knowledge, Attitude, and Practice on Pediatric Tuberculosis Management among Healthcare Workers in the Centre Region of Cameroon: A Cross-Sectional Study. J Trop Med. 2022;2022:4482131.
