# Supplementary material for: Factors contributing to low tuberculosis diagnosis among children aged 0–14 years in Gem Sub County in Siaya County, Kenya
Source: PLoS One. 2026 Mar 6;21(3):e0335326. doi: 10.1371/journal.pone.0335326 (PMC12965594; doi:10.1371/journal.pone.0335326)
Supplement: Attachment — Submitted filename: Response to Reviewers ...docx [file pone.0335326.s004.docx]

**Manuscript Number: PONE-D-25-53931**; **Title: Factors Contributing to Low Tuberculosis Diagnosis Among Children Aged 0-14 Years in Gem Sub County in Siaya County, Kenya.**

We sincerely thank the editor and both reviewers for the constructive, and insightful feedback provided on the manuscript. The comments have greatly helped in improving the clarity and overall quality of the paper.

| **S/N** | **Reviewer comments** | **Correction / Response** |
| --- | --- | --- |
|  | **Reviewer 2 comments within the document** |  |
|  | Introduction: Merge these sentences or rephrase | Merged |
|  | Introduction: Better to speak to either “paediatric” or “child/children/childhood” | Well noted, childhood adopted |
|  | Reference required here, citation inconsistencies? | Additional references/citations added where needed. all in-text citations standardized. |
|  | Please state aims and objectives clearly. | Edited, stated clearly |
|  | Methodology: Cross sectional or mixed study? | Clarified/edited: cross sectional study employing an embedded mixed-methods design, with two strands (quantitative and qualitative component) mixed at design phase |
|  | The sampling technique is unclear. Rephrase, please? | Rephrased and clarified |
|  | There should be a clear demarcation between the quantitative and qualitative data collection process. | Clarified |
|  | Is the questionnaire validated? | Yes clarified, study employed embedded mixed-methods design |
|  | How was the qualitative data collected, transcribed and coded? | Clarified/edited (Data was collected using validated semi structured questionnaires, using the Kobo Collect platform on Android tablets. Both quantitative and qualitative questions in form of addons were collected from all the respondents. |
|  |  |  |
|  | Results: Subheadings required here to differentiating the quantitative from the qualitative aspect of the study. | Thanks, however this was an embedded mixed methods study |
|  | Clarify tables and figures: The labelling of the tables and figures begin from 4.? There is no explanation for some of the figures in the body of the manuscript- Figures 4.2, 4.3, 4.4. Table 4.4, and 4.7 give no additional value to the manuscript. | Table/figure labels and descriptions have been standardized (Table 1, Figure 2). |
|  | Table 6: This question is unclear. Is it testing the knowledge with regards to major signs and symptoms of TB disease?> | Clarified, knowledge with regards to major signs and symptoms |
|  | Discussion: Introduction section is a repetition of the results! | Edited |
|  | Line 339: Is this directing the reader to the author in the reference section?? | Clarified, its directing to the author in reference section, the flow now edited |
|  | Line 345: This sentence is incomplete. | edited |
|  | This is the first time this is featuring, there is a need for consistency, Xpert/MTB RIF or GeneXpert? | Clarified, GeneXpert adopted |
|  | The age range of the study population is 12-18. How will this be of benefit to them? | Edited, children 0-14 years |
|  | **Reviewer comments attached in email** |  |
|  | R1: The topic is highly relevant for public health and aligns with PLOS ONE’s scope. However, the findings mainly reinforce existing knowledge without offering strong new insights beyond local context. The authors should clarify what new contribution their study adds to the broader literature. | The study offers local insights from a high-burden setting, 1. multiple facility visits up to 12, before TB diagnosis, points to the severity of missed opportunities at the primary care level  2. marked differences in diagnosis by HCW cadre, calls for practical guidance/ training interventions in resource disadvantaged settings like Kenya where task-shifting is common.  3. While TB-HIV stigma is widely reported, we provide data showing that over half of caregivers explicitly linked childhood TB to HIV, and sought care only after persistent symptoms.  4 The finding informs TB programming in Kenya’s devolved health system and similar resource-limited settings to adapt TB diagnosis and treatment guidelines to local realities. |
|  | R1: The sampling and data analysis sections are clearly explained but need stronger justification. The sample size (16 caregivers, 71 HCWs) is small for meaningful regression analysis. The authors should explicitly acknowledge this limitation and avoid overinterpretation of odds ratios with extremely wide confidence intervals. Multivariate analysis was omitted due to small sample size this is appropriate but should be discussed as a limitation for causal inference. | Noted and the limitation is acknowledged in strengths and limitation section |
|  | R1: The qualitative quotes are rich and well-selected but should be more clearly integrated with quantitative findings in the discussion to illustrate triangulation of results. | Included/integrated |
|  | The manuscript requires careful English editing to improve flow, avoid redundancy, and correct grammar (e.g., “pediatric population. This is mainly attributed…” should be merged into one sentence). Table formatting and figure captions should follow PLOS ONE guidelines. | Edited where necessary |
|  | Clarify abbreviations at first use (e.g., “HCW,” “TB4 register”). | Clarified. All abbreviations now defined at first use |
|  | Strengthen the conclusion by focusing on actionable recommendations rather than restating results. | Done, conclusions now strengthened with actionable recommendations |
|  | The authors should elaborate more on the methodology used in this manuscript. A mixed study research, like any other research requires clarity. The authors should clearly define the mixed methods approach used and how it helped address their research question. They should describe the study design whether the study followed a convergent, explanatory sequential, exploratory sequential, design and provide a rationale for their choice. | We thank the reviewer for highlighting this point. Upon re-reading, we realize that our description of the qualitative component may have led to a misunderstanding. No separate in-depth interviews were conducted with the 71 healthcare workers. Instead, the study employed an embedded mixed-methods approach; in which qualitative data were collected as addons using open-ended questions within a semi-structured questionnaire. |
|  | R2: Methodology: The sequence of data (quantitative and qualitative) collection should be explained. This will not only improve rigor, but also validity and reliability of the study. It is not stated how the in-depth interviews were carried out among 71 health care workers and how this large volume of data was handled.  The semi structured questionnaire and in-depth interview questions have not been included in the manuscript. | We apologize for the confusion in the original manuscript. No in-depth interviews were conducted with the 71 healthcare workers. The embedded method approach was applied, with two strands (quantitative and qualitative component) mixed at design phase. We have revised the relevant sections for precision |
|  | R3: Results: This section needs to be structured to improve clarity in the mind of the readers and make it more meaningful. Mixed study requires that both qualitative and quantitative results are initially reported separately, not treated as separate entities but rather as complementary sources of evidence.  It is difficult to see where the two data sets were integrated.  The labelling of the tables and figures begin from 4.? There is no explanation for some of the figures in the body of the manuscript- Figures 4.2, 4.3, 4.4. Table 4.4, and 4.7 give no additional value to the manuscript. | The embedded method approach was applied, with two strands (quantitative and qualitative component) mixed at design phase.  Table 4.4 removed as it adds limited value, however 4.7 explains training versus CXR interpretation and sample collection |
|  | Discussion: The discussion starts with a repetition of the results. The authors need to integrate discussion that highlights areas of convergence, divergence, or complementarity of the data sets. In some places, cited articles are substitute by numbers with no context.  There should be a comment or two in the discussion section with regards to this statement- A 33-year-old link assistant noted, “Chest X-rays are not advisable in children due to harmful rays, which may lead to missed cases due to fear.”  The authors would have encountered several biases in this study (e.g. selection, social desirability, sampling, response etc.)- How did the authors mitigate against biases?  The duration of the study (1- month) makes it imprudent to generalize the findings, as thus, should also be noted as a limitation. The factors influencing diagnosis might fluctuate seasonally or be influenced by specific short-term events (e.g. a temporary stock-out) that would not be representative of a typical situation throughout the year. Additionally, seasonal illnesses e.g. flu can mask TB symptoms and contribute to a delay in seeking care.  In general, the discussion section can be improved upon. | The introduction has been edited  Added in discussion section  Limitations acknowledged  Noted and acknowledged  Discussion has been thoroughly revised for depth, flow, and synthesis. |

We believe these revisions fully address the concerns raised and have significantly strengthened the manuscript. We are grateful for the opportunity to improve the work and look forward to your decision.

**Sincerely,**

**Lilian Atieno Okumu** (Corresponding Author)

On behalf of all co-authors

Email: [okumulilian@yahoo.com](mailto:okumulilian@yahoo.com?referrer=grok.com)

Date: December 28, 2025
