# Supplementary material for: Factors contributing to low tuberculosis diagnosis among children aged 0–14 years in Gem Sub County in Siaya County, Kenya
Source: PLoS One. 2026 Mar 6;21(3):e0335326. doi: 10.1371/journal.pone.0335326 (PMC12965594; doi:10.1371/journal.pone.0335326)
Supplement: Attachment — Submitted filename: PONE-D-25-53931R1.docx [file pone.0335326.s003.docx]

Thank you for the opportunity to revise the corrections on the manuscript- **factors that contribute to low tuberculosis diagnosis in children aged 0-14years in Gem County, Siaya County, Kenya**. I commend the authors for adapting the changes advised.

Introduction & Methodology-

- These are clear and easy to understand

Results-

- (Line 186) There is need to specify the group of respondents being referred to. Half 9; 56.3% clearly does not refer to the HCW.
- The message conveyed in Fig 3 is unclear. The authors should tidy up the information on Tables 6 &7 ***OR*** summarise the information on them in prose form and expunge the tables.

Discussion-

- Line 475-6: “CXR are not advisable for children…”- this is HCW misconception and should be moved to facility/HCW factors contributing to delayed diagnosis.
- Line 490-497: “Qualitative insights in skilled deficits…” should also be moved to facility/HCW factors contributing to delayed diagnosis.
- Line 563 “The absence of essential tools, coupled with poor sample collection techniques, was a significant constraint to TB diagnosis” should come under diagnostic constraints.
- Line 558- The sentence here is a repetition as it speaks to HCW technique and should be moved the appropriate section
