# Supplementary material for: Factors contributing to low tuberculosis diagnosis among children aged 0–14 years in Gem Sub County in Siaya County, Kenya
Source: PLoS One. 2026 Mar 6;21(3):e0335326. doi: 10.1371/journal.pone.0335326 (PMC12965594; doi:10.1371/journal.pone.0335326)
Supplement: Attachment — Submitted filename: Response to Reviewer24012026.docx [file pone.0335326.s005.docx]

**Manuscript Number: PONE-D-25-53931**; **Title: Factors Contributing to Low Tuberculosis Diagnosis Among Children Aged 0-14 Years in Gem Sub County in Siaya County, Kenya.**

We sincerely thank the reviewer for the additional insightful feedback provided on the manuscript to improve the clarity and overall quality of the paper.

| **S/N** | **Reviewer comments** | **Correction / Response** |
| --- | --- | --- |
|  | **Reviewer 2 comments within the document** |  |
|  | RESULTS  -(Line 186) There is need to specify the group of respondents being referred to. Half 9; 56.3% clearly does not refer to the HCW.  -The message conveyed in Fig 3 is unclear. The authors should tidy up the information on Tables 6 &7 OR summarise the information on them in prose form and expunge the tables. | Specified, about half of HCWs were female (38, 53.5%), while over half of other respondents (9, 56.3%) were aged 35 years and above.  Figure 3 has been removed from the manuscript as it conveyed limited additional value  Tables 6& 7 combined into one for clarity. Information presented in table 5 |
|  | DISCUSSION  -Line 475-6: “CXR are not advisable for children…”- this is HCW misconception and should be moved to facility/HCW factors contributing to delayed diagnosis.  -Line 490-497: “Qualitative insights in skilled deficits…” should also be moved to facility/HCW factors contributing to delayed diagnosis.  -Line 563 “The absence of essential tools, coupled with poor sample collection techniques, was a significant constraint to TB diagnosis” should come under diagnostic constraints.  -Line 558- The sentence here is a repetition as it speaks to HCW technique and should be moved the appropriate section | Line 475 moved to facility/HCW factors contributing to delayed diagnosis.  Line 490 (qualitative insights) moved as appropriate  Line 563 concern is acknowledged. However, considered as facility/HCW factors  Line 558, moved to appropriate section |
|  | Please upload a copy of Figure 4 which you refer to in your text on page 21. Or if the figure is no longer to be included as part of the submission please remove all reference to it within the text. | Sorry for this. We were referring to figure 1. We have since updated 4 to 1. |

We believe these revisions fully address the concerns raised and have significantly strengthened the manuscript. We are grateful and look forward to your decision.

**Sincerely,**

**Lilian Atieno Okumu** (Corresponding Author)

On behalf of all co-authors

Email: [okumulilian@yahoo.com](mailto:okumulilian@yahoo.com?referrer=grok.com)

Date: January 24, 2026
